# The Antifungal Plant Defensin HsAFP1 Is a Phosphatidic Acid-Interacting Peptide Inducing Membrane Permeabilization

**DOI:** 10.3389/fmicb.2017.02295

**Published:** 2017-11-21

**Authors:** Tanne L. Cools, Kim Vriens, Caroline Struyfs, Sara Verbandt, Marcelo H. S. Ramada, Guilherme D. Brand, Carlos Bloch, Barbara Koch, Ana Traven, Jan W. Drijfhout, Liesbeth Demuyser, Soňa Kucharíková, Patrick Van Dijck, Dragana Spasic, Jeroen Lammertyn, Bruno P. A. Cammue, Karin Thevissen

**Affiliations:** ^1^Centre of Microbial and Plant Genetics, KU Leuven, Leuven, Belgium; ^2^Department of Plant Systems Biology, VIB, Ghent, Belgium; ^3^Graduate Program in Genomic Sciences and Biotechnology, Catholic University of Brasilia, Brasilia, Brazil; ^4^Mass Spectrometry Laboratory, Embrapa Genetic Resources and Biotechnology, Brasilia, Brazil; ^5^Chemistry Institute, Campus Darcy Ribeiro, University of Brasilia, Brasilia, Brazil; ^6^Department of Biochemistry and Molecular Biology, Monash University, Clayton, VIC, Australia; ^7^Department of Immunohematology and Blood Transfusion, Leiden University Medical Center, Leiden, Netherlands; ^8^Laboratory of Molecular Cell Biology, KU Leuven, Leuven, Belgium; ^9^VIB-KU Leuven Center for Microbiology, Leuven, Belgium; ^10^BIOSYST-MeBioS, KU Leuven, Leuven, Belgium

**Keywords:** plant defensins, yeast, antifungal mode of action, lipid membrane target, peptide internalization, membrane permeabilization

## Abstract

HsAFP1, a plant defensin isolated from coral bells (*Heuchera sanguinea*), is characterized by broad-spectrum antifungal activity. Previous studies indicated that HsAFP1 binds to specific fungal membrane components, which had hitherto not been identified, and induces mitochondrial dysfunction and cell membrane permeabilization. In this study, we show that HsAFP1 reversibly interacts with the membrane phospholipid phosphatidic acid (PA), which is a precursor for the biosynthesis of other phospholipids, and to a lesser extent with various phosphatidyl inositol phosphates (PtdInsP’s). Moreover, via reverse ELISA assays we identified two basic amino acids in HsAFP1, namely histidine at position 32 and arginine at position 52, as well as the phosphate group in PA as important features enabling this interaction. Using a HsAFP1 variant, lacking both amino acids (HsAFP1[H32A][R52A]), we showed that, as compared to the native peptide, the ability of this variant to bind to PA and PtdInsP’s is reduced (≥74%) and the antifungal activity of the variant is reduced (≥2-fold), highlighting the link between PA/PtdInsP binding and antifungal activity. Using fluorescently labelled HsAFP1 in confocal microscopy and flow cytometry assays, we showed that HsAFP1 accumulates at the cell surface of yeast cells with intact membranes, most notably at the buds and septa. The resulting HsAFP1-induced membrane permeabilization is likely to occur after HsAFP1’s internalization. These data provide novel mechanistic insights in the mode of action of the HsAFP1 plant defensin.

## Introduction

Plant defensins are peptides (45–54 amino acids), which are suggested to be part of the immune system of plants. They possess a broad-spectrum antimicrobial activity (Carvalho Ade and Gomes, 2009, 2011; De Coninck et al., 2013), but are in general non-toxic for plant or human cells (Thevissen et al., 2007; Tavares et al., 2008; Vriens et al., 2015). Plant defensins are highly structured peptides that exhibit a common fold consisting of a cysteine-stabilized αβ-motif (Van der Weerden and Anderson, 2013) and possess abundant positively charged and hydrophobic amino acids. In the past decades, the antifungal mode of action of various plant defensins has been studied (as reviewed by Vriens et al., 2014). Plant defensins such as NaD1 (Poon et al., 2014), TPP3 (Baxter et al., 2015) and MtDef4 (Sagaram et al., 2013), isolated from tobacco, tomato and barrel clover, respectively, interact with fungal phospholipids, whereas others such as RsAFP2 (Thevissen et al., 2004), Psd1 (de Medeiros et al., 2010) and DmAMP1 (Thevissen et al., 2000, 2003), isolated from radish, pea and dahlia, respectively, interact with fungal-specific sphingolipids like glucosylceramides and mannosyl diinositolphosphoryl ceramides in the membranes of susceptible fungi. Upon interaction with their respective fungal membrane lipid, plant defensins can either be internalized into the fungal cells, thereby affecting intracellular targets in the cytosol or nucleus as in case of MtDef4 (Sagaram et al., 2013), NaD1 (van der Weerden et al., 2010) and Psd1 (Lobo et al., 2007), or remain on the outside of the fungal cells, as in case of RsAFP2 (Thevissen et al., 2012). For NaD1 (van der Weerden et al., 2008), PvD1 (from bean) (Mello et al., 2011), DmAMP1 (Aerts et al., 2006), HsAFP1 (from coral bells) (Aerts et al., 2011), Lp-Def1 (from cream nut) (Vieira et al., 2015) and RsAFP2 (Aerts et al., 2007, 2009) it has been reported that they subsequently induce the production of reactive oxygen species (ROS) and/or apoptosis at relatively low doses, while membrane permeabilization occurs at high concentrations (Thevissen et al., 1999; Bleackley et al., 2014). The latter is suggested to be a non-specific secondary effect rather than the primary cause of microbial killing (Thevissen et al., 1996).

HsAFP1, a plant defensin isolated from the seeds of coral bells (*Heuchera sanguinea*), can inhibit a broad range of fungi, including the model yeast *Saccharomyces cerevisiae* and the most common human pathogen *Candida albicans* (Osborn et al., 1995; Thomma et al., 2002; Thevissen et al., 2007). This peptide is not toxic to human cells (Vriens et al., 2015) and is characterized by a low *in vitro* frequency of spontaneous occurrence of resistance (Thevissen et al., 2007). Moreover, it can inhibit *C. albicans* biofilm formation (Vriens et al., 2015), which points to its potential for further drug development. In view of the latter, it is important to understand its mechanism of action, and in particular to identify its fungal interaction partner(s) and downstream signaling pathways resulting in fungal growth inhibition. Previously, we have demonstrated that HsAFP1 interacts with high-affinity binding sites on membranes of susceptible fungi (Kd = 29 nM) (Thevissen et al., 1997). This interaction is specific, competitive, reversible and saturable, pointing to a specific receptor-ligand interaction. However, the target in the fungal membrane for HsAFP1 remained elusive as, in contrast to RsAFP2, Psd1 and DmAMP1, fungal-specific sphingolipids appear not to be involved in HsAFP1’s mode of action (Aerts et al., 2011).

In the present study, a lipid binding partner of HsAFP1 was identified, after which the domains of both HsAFP1 and the lipid species involved in this interaction were characterized. Internalization of HsAFP1 was investigated in *S. cerevisiae*, as well as HsAFP1-induced membrane permeabilization resulting in cell death.

## Materials and Methods

### Strains and Reagents

In this study, the yeast *Saccharomyces cerevisiae* strain BY4741 and the filamentous fungal pathogen *Fusarium culmorum* K0311 were used. Yeasts were cultured at 30°C in YPD (yeast extract (10 g/L), peptone (20 g/L) and glucose (20 g/L) or YPD/PDB (potato dextrose broth (19.2 g/L), yeast extract (2 g/L), peptone (4 g/L) and glucose (4 g/L)) adjusted to pH7 with 50 mM HEPES, while filamentous fungi were cultured at 22°C in PDB (12 g/L). The plant defensins HsAFP1 and HsAFP1[H32A][R52A] were recombinantly produced in *Pichia pastoris* and purified using the protocol described by Vriens et al. (2015). Linear HsAFP1-derived peptide fragments (HsLin’s) were synthesized and purified as described previously by Goblyos et al. (2013). Cysteine residues were replaced by α-aminobutyric acid (α-ABA; X) to avoid disulphide bond formation in the linear peptides.

All lipids and PIP Strips were purchased from Echelon (Salt Lake City, UT, United States), except for dimyristoylphosphatidylcholine (PC), dimyristoylphosphatidylglycerol (PG), dimyristoylphosphatidic acid (PA) used for the DSC experiments and methyl-PA (Avanti Polar Lipids, Alabaster, AL, United States). rELISA assays were performed in Nunc-Immuno TM plates Polysorp. Bovine serum albumin (BSA), NaH_2_PO_4_.2H_2_O and Tween 20 were used for buffer preparations, while 4-nitrophenyl phosphate (4-NPP) disodium salt, 5-bromo-4-chloro-3-indolyl-phosphate (BCIP) and nitro blue tetrazolium (NBT) were used as enzyme substrates in color reactions for quantification. Antiserum, raised in rabbits injected with HsAFP1 [as was previously done for antisera against the plant defensins RsAFP2 and DmAMP1 (Francois et al., 2002)], and anti-rabbit IgG-alkaline phosphatase were used respectively as primary and secondary antibody. For HsAFP1 internalization and membrane permeabilization studies, propidium iodide (PI), 1-ethyl-3-(3-dimethylaminopropyl) carbodiimide (EDC) and BODIPY-FL-EDA were used.

### PIP Strip Overlay Assay for HsAFP1 Lipid Binding Partner Identification

HsAFP1 lipid binding partners were determined via lipid overlay assay by using PIP Strips, containing 100 pmol spots of all phospholipids. The manufacturer’s instructions were adjusted as follows (i) 1.7 μM HsAFP1 and BCIP/NBT were used as lipid interaction partner and enzyme (alkaline phosphatase (AP)) substrate, respectively, (ii) the enzymatic color reaction was performed in alkaline phosphate buffer pH9.5 with BCIP (0.2 g/L) and NBT (0.3 g/L) as substrate and (iii) after 5 min this reaction was stopped with an EDTA (6 μg/L) solution. As positive controls, 1 μL of HsAFP1 (16.8 μM) or secondary antibodies (1/20 diluted) were spotted separately on top of the PIP Strips (not on the control membrane). When the spots were dry, the lipid overlay assay was performed as described above. HsAFP1-lipid binding was determined via pixel intensity quantification of every spot via Image Studio Lite software.

### Reverse ELISA (rELISA) Assay for Quantification of HsAFP1 or HsAFP1[H32A][R52A] Binding

HsAFP1- or HsAFP1[H32A][R52A]-lipid binding was quantified in a rELISA assay, adapted from the protocol described by Thevissen et al. (2004). First, the lipid solution (8 μg/mL; dissolved in methanol) was dried overnight (ON) at room temperature in Polysorp ELISA plates. All subsequent steps were performed at 37°C. After lipid coating, a blocking step with 3% BSA in phosphate-buffered saline (PBS) pH7.4 for 2–3 h was performed. Next, a two-fold dilution series of HsAFP1 was prepared in 10% blocking buffer (or in 0.2 M – 0.0125 M phosphate buffers for phosphate binding experiments) and added to the rELISA wells for 1.5 h. Subsequently, the wells were incubated with primary antibody (1/3000 diluted; 1 h), secondary antibody (1/3000 diluted; 1 h), and 4-NPP as AP substrate (1 mg/mL; 10 min). Absorbance at 405 nm was used as read out. For the rELISA assays, the highest values of the negative controls, samples without HsAFP1, were used as background signal and subtracted from the samples. For all competitive rELISA assays, two-fold dilution series of HsAFP1 in the absence or presence of a four-fold excess of the competitor (compared to the highest concentration of HsAFP1) was added to the rELISA wells in the peptide binding step. In these experiments, the values of the negative controls, samples with 4x competitor, were used as background signal. For all rELISA assays, the % of HsAFP1-lipid binding was calculated relative to the HsAFP1-PA signal obtained at the highest HsAFP1 concentration (12.5 μM).

### Lipid Vesicles

Two types of vesicles were prepared using following molar ratios of the phospholipids: PC/PG 75/25 (Payne et al., 2016) and PC/PG/PA 75/15/10. A total of 20 mg lipid powder was dissolved in chloroform/methanol 2/1 and subsequently dried as a lipid film using a rotavap and 3 h of vacuum drying. Next, the lipid film was dissolved in 2-3 mL of 50 mM HEPES pH7 (Payne et al., 2016) + 0.005 M NaCl.

### Differential Scanning Calorimetry (DSC)

Thermograms were obtained using a VP-DSC (MicroCal Inc., Northampton, MA, United States) device as described by Brand et al. (2012), except for the scanning rate (0.5°C/min) and the peptide concentration (40 μM).

### Fluorescent Labeling of HsAFP1 with the Fluorophore BODIPY

HsAFP1 was fluorescently labeled via its carboxyl groups with the fluorophore BODIPY-FL-EDA as described by van der Weerden et al. (2010).

### Fungal Growth Inhibition Assay

The antifungal activity of HsAFP1[H32A][R52A] and BODIPY-HsAFP1 against *F. culmorum* were determined as described by Vriens et al. (2015).

### Minimum Fungicidal Concentration (MFC)

Exponentially growing *S. cerevisiae* cells in YPD were incubated (OD_600_ = 1) in PDB/YPD with HsAFP1 or with HsAFP1[H32A][R52A] for 150 min. Both at the start and at the end of the treatment, surviving yeast cells were determined via plating assays. In a plating assay, 10-fold dilution series of yeast cells in PBS were prepared, after which 100 μL was plated on YPD plates. After 2 days of incubation at 30°C, the number of colony forming units (CFU) was counted and cell death was calculated relative to time zero (t0). MFC50 values indicating the MFC resulting in 50% cell death, were determined.

### Cell Treatment for Confocal Microscopy or Flow Cytometry Experiments

Exponentially growing yeast cells in YPD were incubated (OD_600_ = 1) in PDB/YPD with HsAFP1 or with BODIPY-HsAFP1 (B-Hs; 4 – 48 μM), propidium iodide (PI) (2 μg/mL) for 150 min (endpoint) unless stated otherwise. In parallel to confocal microscopy or flow cytometry experiments, plating assays were performed to determine MFC50 values, as described above.

### Confocal Microscopy of *Saccharomyces cerevisiae* Cells Treated with BODIPY-HsAFP1

HsAFP1 localization studies were performed on yeast cells treated with BODIPY-HsAFP1 (4, 16, or 48 μM) and PI (2 μg/mL) as described above. After 60–150 min of treatment, cells were visualized by confocal microscopy with the FluoView FV1000 confocal microscope (Olympus IX81) and its software. We used a 60x magnification objective and 1-6x computer zoom. The 488 nm laser line of the Argon laser was used for visualization of BODIPY and the 559 nm laser for PI. To ensure that there is no spectral overlap between the green and red channel of our microscope, we examined fluorescence of BODIPY-HsAFP1 and PI in both channels and only observed a fluorescent signal for BODIPY-HsAFP1 in the green channel and a fluorescent signal for PI in the red channel and not vice versa. Hence, no cross-talk between the channels was found in our experimental setup.

### Flow Cytometry of *Saccharomyces cerevisiae* Cells Treated with BODIPY-HsAFP1

HsAFP1 uptake and HsAFP1-mediated membrane permeabilization in yeast cells were determined via flow cytometric analysis. Yeast cells were treated with BODIPY-HsAFP1 (B-Hs) and PI, as described above, and subjected to flow cytometry on a BD Influx^TM^ cell sorter. Approximately 10,000 cells were monitored for fluorescence at 530/40 nm (FL2_λ_ex_ = 488 nm) and 610/20 nm (FL11_ λ_ex_ = 561 nm) for the detection of BODIPY-HsAFP1 uptake (B-Hs+) and membrane permeabilization (PI+), respectively. Data from cells treated with MQ were used as background signal. For the kinetic experiments in **Figure 8**, the cell cultures were divided in 4 subpopulations (B-Hs-/PI-, B-Hs+/PI-, B-Hs-/PI+ and B-Hs+/PI+) based on the fluorescence intensities of the control (MQ) treatment, which was B-Hs-/PI-.

### Data Analysis

Data were analyzed with GraphPad Prism 6 and mean ± standard error of mean (SEM) were represented for *n* ≥ 2 experiments. We assumed that all data are normally distributed. To analyze significant differences between the reference HsAFP1-PA binding (at 12.5 μM HsAFP1) and HsAFP1 bindings in other conditions, unpaired student *t*-test with Welch’s correction (if only two conditions were compared) or one-way ANOVA followed by Dunnett multiple comparison (if more than two conditions were compared) was performed. ^∗^, ^∗∗^, ^∗∗∗^ and ^∗∗∗∗^ represent *P* < 0.05, *P* < 0.01, *P* < 0.001 and *P* < 0.0001, respectively. Data of the thermal scans (DSC experiments) were normalized for the vesicle concentration, baseline subtracted (linear connect) and subsequently fitted with a non-two state model using the MicroCal Origin^TM^ software. To determine significant differences between MIC50 values of native and BODIPY-labeled HsAFP1, an unpaired student *t*-tests was used. Flow cytometric data were presented as means ± SEM of the % of (BODIPY-HsAFP1 or PI) positive cells relative to the whole cell population. One-way ANOVA with Dunnett multiple comparison was used to determine significant differences in the size of the subpopulations compared to those at the lowest BODIPY-HsAFP1 concentration or time zero.

## Results

### HsAFP1 Interacts with Phospholipids

**FIGURE 1:**
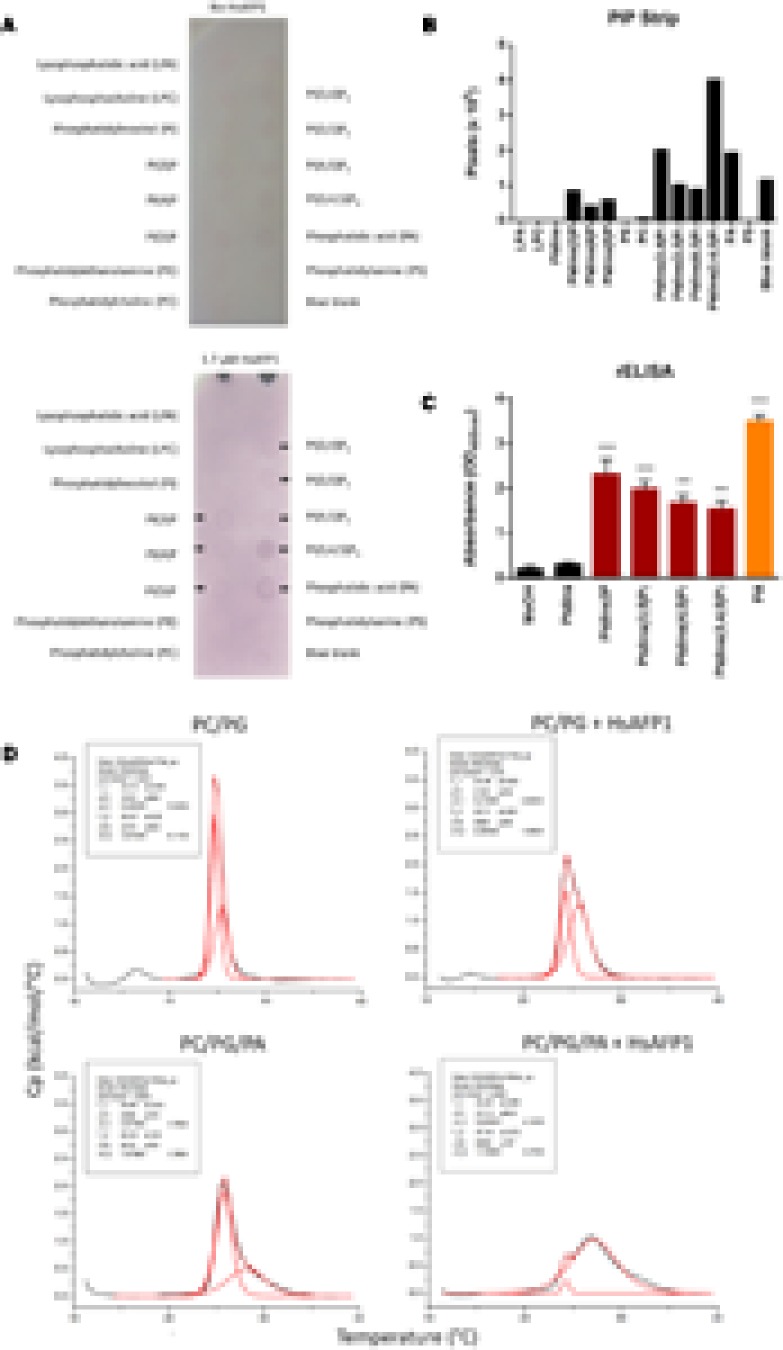
Lipid interaction partners of HsAFP1 determined via **(A,B)** protein-lipid overlay assay using PIP Strips, **(C)** rELISA and **(D)** phospholipid vesicles. **(A)** The PIP Strips were incubated with HsAFP1 (lower membrane) or without HsAFP1 (top control membrane) and subsequently with HsAFP1 antiserum, anti-rabbit IgG-Alkaline Phosphatase and 4-nitrophenyl phosphate. Colored spots represent HsAFP1 binding events, as indicated by black arrows **(A)**, which were quantified via Image Studio Lite **(B)**. **(C)** Optical density (OD_405__nm_) values representing the interaction of 12.5 μM HsAFP1 with the control (MeOH) and all tested phospholipids. Data are means ± SEM, for *n* ≥ 3 experiments. Significant differences between HsAFP1-MeOH and HsAFP1-lipid interactions were determined via one-way ANOVA followed by Dunnett multiple comparison, with ^∗∗∗^ and ^∗∗∗∗^ representing *P* < 0.001 and *P* < 0.0001, respectively. **(D)** Thermograms of phosphatidylcholine(PC)/phosphatidylglycerol(PG) (75/25) and PC/PG/phosphatidic acid(PA) (75/15/10) vesicles in absence or presence of HsAFP1, as determined via DSC experiments. Representative graphs of the phase transition of 0.5 mM liposomes in absence or presence of 40 μM HsAFP1 are presented in black, with the corresponding fit (using a non-two state model) shown in red. Both the fit of the two peaks as well as the overall fit were presented in red. Phase transition temperatures of both peaks of the fit (Tm1 and Tm2) ± SD are specified in the box.

We assessed potential binding between HsAFP1 and specific lipids using PIP Strips, containing phosphoinositides and other biologically important lipids. HsAFP1 bound to PA and several phosphatidylinositol phosphate (PtdInsP)-derivatives, including PtdIns(3)P, PtdIns(4)P, PtdIns(5)P, PtdIns(3,4)P_2_, PtdIns(3,5)P_2_, PtdIns(4,5)P_2_ and PtdIns(3,4,5)P_3_ (**Figures 1A,B**). This was further confirmed using reverse ELISA (rELISA), in which lipids are immobilized to the wells of microtiter plates and bound HsAFP1 is detected immunologically. This type of ELISA was previously used to characterize the binding of RsAFP2 to fungal glucosylceramides (GlcCer) (Thevissen et al., 2004). Using rELISA assays, we assessed HsAFP1 binding to the above indicated lipid species, except for PtdIns(3,4)P_2_. Consistent with the PIP Strip overlay assay, HsAFP1 bound to PA (orange bar in **Figure 1C**) as well as to several PtdInsP-derivatives containing at least one phosphate (P) group (red bars in **Figure 1C**). However, binding of HsAFP1 to PA was most pronounced and therefore further investigated. The PIP Strip and rELISA data on HsAFP1-PA interactions were further confirmed using lipid vesicles. Using Differential Scanning Calorimetry (DSC), the thermotropic behavior of these vesicles in the absence and presence of HsAFP1 was determined. Using a non-two state fitting model, two phase transition temperatures (T_m_1 and T_m_2) were determined. Shifts in T_m_ values are indicative for membrane interactions. We observed small T_m_1 and T_m_2 shifts (ranging between 0.3 and 0.4°C) for the phosphatidylcholine (PC)/phosphatidylglycerol (PG) (75/25) vesicles upon addition of HsAFP1, while the T_m_1 and T_m_2 shifts (ranging between 1.0 and 1.6°C) were larger in case of the PA-containing PC/PG/PA (75/15/10) vesicles (**Figure 1D**). Together, these results point to a HsAFP1-PA interaction in non-membrane (as in the rELISA assays) as well as in membrane (lipid bilayer) conditions.

### HsAFP1 Interacts with the Phosphate Group of Phosphatidic Acid via Its Most C-terminal Arginine

**FIGURE 2:**
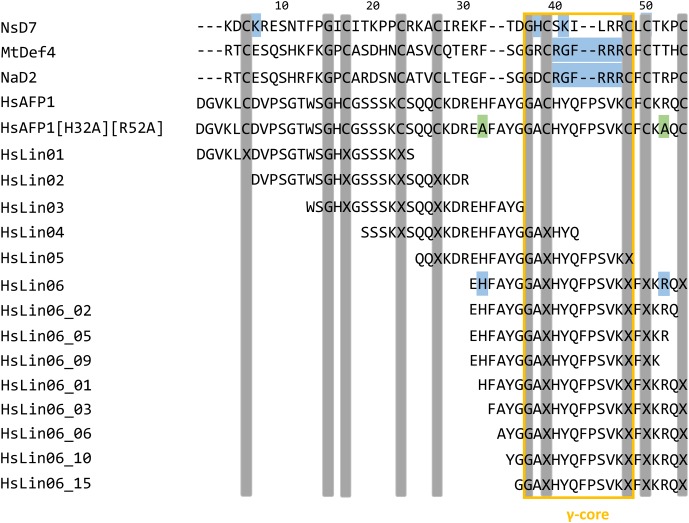
Amino acid sequence alignment of HsAFP1 with HsAFP1 mutant HsAFP1[H32A][R52A], HsAFP1-derived peptide fragments (HsLin01-HsLin06), HsLin06-derived peptide fragments (HsLin06_02;05;09;01;03;06;10 and 15) and the plant defensins NsD7, MtDef4 and NaD2. Gray bars indicate the highly conserved amino acids among plant defensins. The blue, green, and the orange boxes represent the position of the PA binding domain, the amino acid substitutions and the γ-core, respectively. (-) denote gaps in the alignment.

**FIGURE 3:**
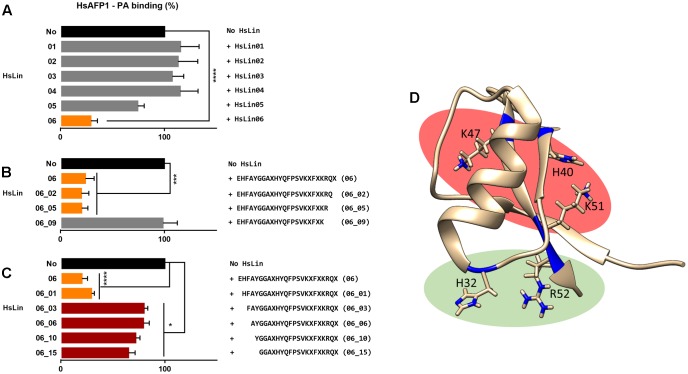
Amino acids of HsAFP1 important for phosphatidic acid (PA) binding, using competitive rELISA assays. Competition of 12.5 μM HsAFP1 with 50 μM HsLin01-06 **(A)**, C-truncated HsLin06 variants (HsLin06_02;05;09 in **Figure 2**; **(B)** or N-truncated variants (HsLin06_01;03;06;10;15 in **Figure 2**; **(C)** with both peptides co-incubated. Data are means ± SEM, for *n* ≥ 3 experiments. Data are expressed relative to the HsAFP1-PA binding without HsLin. Significant differences between HsAFP1 and HsAFP1 + 4x HsLin interactions were determined via one-way ANOVA followed by Dunnett multiple comparison, with ^∗^, ^∗∗∗^, and ^∗∗∗∗^ representing *P* < 0.05, *P* < 0.001, and *P* < 0.0001, respectively. **(D)** 3D structure of HsAFP1 with the positively charged amino acids [histidine (H) 32 and 40, lysine (K) 47, K51 and arginine (R) 52] of HsLin06 drawn in blue on its backbone, using the Chimera visualization program. Green clustered amino acids (H32 and R52) are important for PA binding while the red clustered (H40, K47 and K51) are not.

**FIGURE 4:**
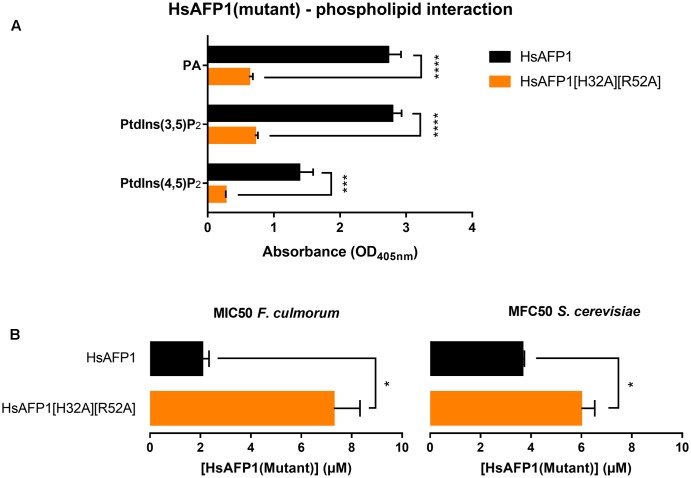
Reduced phospholipid binding capacity **(A)** and antifungal activity **(B)** of HsAFP1[H32A][R52A] compared to native HsAFP1. Data are means ± SEM, for *n* ≥ 3 experiments. **(A)** Optical density (OD_405__nm_) values representing the interaction of 12.5 μM HsAFP1 or HsAFP1[H32A][R52A] with all phospholipids tested [phosphatidic acid (PA), phosphatidylinositol(3,5)bisphosphate (PtdIns(3,5)P_2_), phosphatidylinositol(4,5)bisphosphate (PtdIns(4,5)P_2_)], determined via rELISA assays. Significant differences between HsAFP1 and HsAFP1[H32A][R52A] were determined via two-way ANOVA followed by Sidak multiple comparison, with ^∗∗∗^ and ^∗∗∗∗^ representing *P* < 0.001 and *P* < 0.0001, respectively. **(B)** Minimum Inhibitory Concentration of HsAFP1 or HsAFP1[H32A][R52A] resulting in 50% cell growth reduction (MIC50) of *F. culmorum* and Minimum Fungicidal Concentration of HsAFP1 or HsAFP1[H32A][R52A] resulting in 50% cell death (MFC50) of *S. cerevisiae*. Significant differences between HsAFP1 and HsAFP1[H32A][R52A] were determined via an unpaired student *t*-test with Welch’s correction, with ^∗^ representing *P* < 0.05.

To identify the HsAFP1 domain responsible for the binding to PA, we tested competition for PA binding between HsAFP1 and different linear 24-mer HsAFP1-derived peptide fragments spanning the entire HsAFP1 sequence (HsLin01-06; **Figure 2**). HsAFP1 was co-incubated with an excess of these linear HsAFP1-derived fragments and the amount of HsAFP1 bound to PA in the presence of the linear fragments was quantified via rELISA. As the HsAFP1 antiserum interacted to a limited extent with various HsLin peptides, these background values were subtracted from the corresponding data. We found that only HsLin06 (*P* < 0.0001) significantly reduced the binding of HsAFP1 to PA, indicating that the C-terminal part of HsAFP1 is important for PA binding (orange bar in **Figure 3A**). Additionally, we performed a sequential competitive rELISA in which HsAFP1 was incubated with PA for 30 min prior to the addition of an excess of HsLin06, allowing HsAFP1 to bind to PA first. We found that bound HsAFP1 could be expelled by excess of HsLin06 (Supplementary Figure S1) and this to a similar extent as in a simultaneous rELISA assay (**Figure 3A**), indicating that the binding of HsAFP1 to PA is reversible. To further identify the domains of HsLin06 important for PA binding, we assessed competition for PA binding between HsAFP1 and HsLin06 variants that were incrementally shortened at their N-terminus (HsLin06_01;03;06;10;15; **Figure 2**) or C-terminus (HsLin06_02;05;09; **Figure 2**). Only C-terminally shortened HsLin06 variants 02 (*P* = 0.0002) and 05 (*P* = 0.0002) could compete with HsAFP1 for PA binding, and this to the same extent as HsLin06 (around 75–80%; orange bars in **Figure 3B**), while variant HsLin06_09 could not compete at all (*P* = 0.9998; gray bar **Figure 3B**). Since HsLin06_02 and 05 differ from HsLin06_09 only in their C-terminal arginine (R, position 52 in HsAFP1), these data show that R52 is an important amino acid for PA binding. Of the HsLin06 variants shortened at their N-terminus, only HsLin06_01 (*P* < 0.0001) could compete with HsAFP1 for PA binding, and this to the same extent as HsLin06 (around 75–80%; orange bars **Figure 3C**), while all other variants (HsLin06_06;10;15) could only partially (around 35%; red bars in **Figure 3C**) do so (with *P* = 0.0229, *P* = 0.0024 and *P* = 0.0003, respectively). Hence, it seems that the N-terminal histidine of HsLin06 (H, position 32 in HsAFP1) is important, but not essential for PA binding as HsLin06 variants 03; 06; 10 and 15, which are all lacking this amino acid, can still partially compete with HsAFP1 for PA binding. To confirm the important role of H32 and R52 for HsAFP1’s PA binding action, a HsAFP1 mutant peptide in which both H32 and R52 were replaced by alanine (A) was produced (HsAFP1[H32A][R52A] (**Figure 2**)) and subsequently tested for PA binding and antifungal activity. Binding of HsAFP1[H32A][R52A] to PA was reduced by 77% as compared to native HsAFP1 (**Figure 4A**). Also binding of HsAFP1[32A][R52A] to PtdIns(3,5)P_2_ and PtdIns(4,5)P_2_ was significantly reduced compared to native peptide (**Figure 4A**). Next, the MIC50 (minimum inhibitory concentration resulting in 50% cell growth reduction) on *F. culmorum* and the MFC50 (MFC resulting in 50% cell death) on *S. cerevisiae* were determined for HsAFP1[H32A][R52A] and native HsAFP1. Compared to the native peptide, the MIC50 and the MFC50 of HsAFP1[H32A][R52A] were 3.5-fold and 2-fold higher (**Figure 4B**), respectively, demonstrating reduced antifungal activity as well as reduced phospholipid (PA/PtdIns) binding for HsAFP1[H32A][R52A].

**FIGURE 5:**
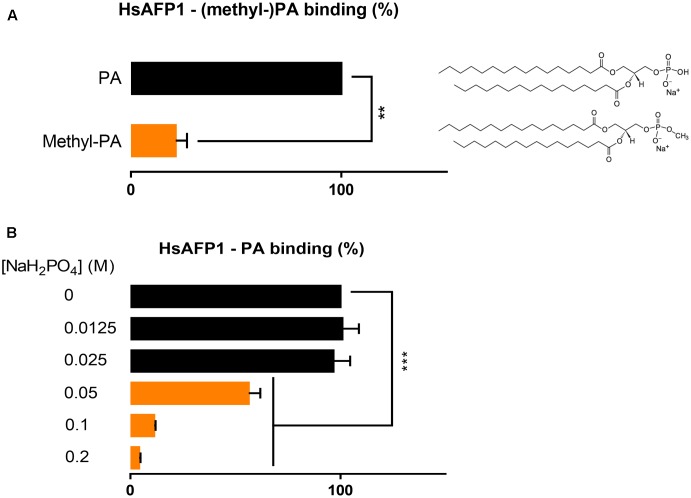
Phosphate-dependent binding of HsAFP1 to phosphatidic acid (PA), determined via rELISA assays. Interaction of 12.5 μM HsAFP1 with PA or methyl-PA **(A)** or for the interaction of HsAFP1 with PA in buffers with different phosphate concentrations **(B)**. Data are means ± SEM, for *n* ≥ 3 experiments. Data are expressed relative to the HsAFP1-PA binding **(A)** or relative to the HsAFP1-PA binding in demi-water (0 M NaH_2_PO_4_) (dotted line) **(B)**. Significant differences between PA and methyl-PA interactions were determined via an unpaired student *t*-test with Welch’s correction **(A)**, while significant differences between demi-water (0M NaH_2_PO_4_) and phosphate buffers were determined via one-way ANOVA followed by Dunnett multiple comparison **(B)**, with ^∗∗^ and ^∗∗∗^ representing *P* < 0.01 and *P* < 0.001, respectively.

Next, we investigated structural requirements of PA enabling HsAFP1 binding, focusing on the phosphate group in PA. We investigated the binding of HsAFP1 to methyl-PA in which the hydroxyl group of the phosphate group of PA is methylated, resulting in a changed ionization state of PA and in a less accessible phosphate group. Binding of HsAFP1 to methyl-PA was significantly reduced as compared to PA (**Figure 5A**; *P* = 0.0046), indicating that a free phosphate group in PA is important for HsAFP1 binding. Next, we assessed binding of HsAFP1 to PA in buffers with different phosphate concentrations. The higher the phosphate concentration of the buffer, the lower the amount HsAFP1 bound to PA was measured (**Figure 5B**). This result indicates competition between PA and free phosphates for HsAFP1 binding. Together, these data point to the phosphate group in PA as the HsAFP1 binding feature.

### HsAFP1 Is Accumulating at the Yeast Cell Surface and Induces Membrane Permeabilization

Given HsAFP1’s capacity to bind membrane phospholipids, we investigated potential membrane-dependent localization of HsAFP1 in yeast cells. To this end, we labeled HsAFP1 with the green fluorescent marker BODIPY, as previously described for NaD1 (van der Weerden et al., 2010) and confirmed that the label did not affect the antifungal activity of HsAFP1 nor its PA binding (Supplementary Figure S2).

**FIGURE 6:**
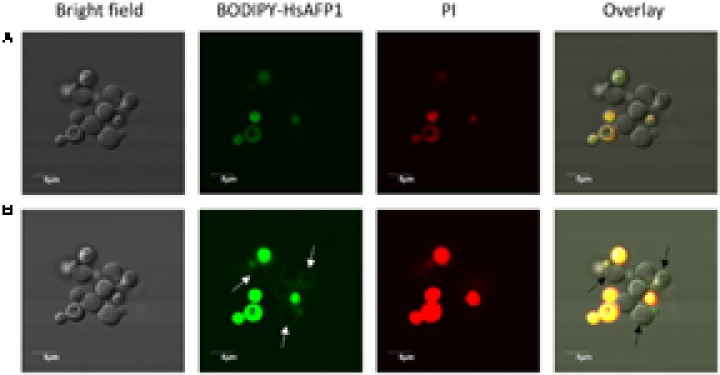
HsAFP1 localization in *S. cerevisiae* cells treated with high (48 μM) BODIPY-HsAFP1 concentrations. Confocal microscope images of 150 min-treated *S. cerevisiae* cultures with 48 μM BODIPY-HsAFP1. Propidium iodide (PI; 2 μg/mL) was used to identify membrane permeabilization. **(A)** Confocal images acquired with normal laser intensities [Alexa fluor 488-10% (argon laser)-HV451 and PI-1% (559nm laser)-HV405]. **(B)** Confocal images taken at high laser intensities [Alexa fluor 488-10% (argon laser)-HV573 and PI-1% (559 nm laser)-HV566], without impacting autofluorescence. Representative BODIPY-HsAFP1+/PI- cells are indicated with arrows. Bar: 5 μm.

At high (10x HsAFP1’s MFC50; 48 μM) BODIPY-HsAFP1 doses and normal laser intensities (**Figure 6A**), most yeast cells accumulated BODIPY-HsAFP1 intracellularly after 150 min of incubation, pointing to HsAFP1 uptake. To assess whether the latter is a result of HsAFP1-induced membrane permeabilization, which was reported at high HsAFP1 doses (>8.4 μM under different experimental conditions) (Thevissen et al., 1999), we simultaneously assessed membrane permeabilization via (red fluorescent) PI (Riccardi and Nicoletti, 2006) and found that the yeast cells that accumulated BODIPY-HsAFP1 intracellularly had permeabilized membranes (BODIPY-HsAFP1+/PI+). However, some cells (<1% of the BODIPY-HsAFP1 + population, as measured via flow cytometric analysis) showed intracellular HsAFP1 accumulation and intact membranes (BODIPY-HsAFP1+/PI-; as indicated by arrows in Supplementary Figure S3). Additionally, at higher laser intensities, a second subpopulation of BODIPY-HsAFP1+/PI- cells were apparent that accumulated BODIPY-HsAFP1 on their cell surface and had intact membranes (as indicated by arrows in **Figure 6B**). At this laser intensity no autofluorescence of the cells was visible (data not shown). Hence, at 48 μM BODIPY-HsAFP1, there seem to be two distinct subpopulations with intact membranes that either accumulate BODIPY-HsAFP1 on their surface (which are characterized by low intensity green fluorescence and hence, only apparent when using high laser intensity) or accumulate BODIPY-HsAFP1 intracellularly (characterized by higher intensity green fluorescence and hence, apparent when using normal laser intensity).

**FIGURE 7:**
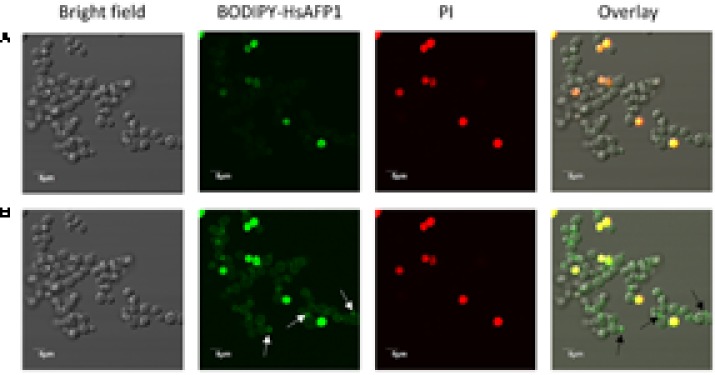
BODIPY-HsAFP1 is located intracellularly in membrane-permeabilized cells and at the cell surface of non-membrane permeabilized *S. cerevisiae* cells treated with 16 μM BODIPY-HsAFP1. Confocal microscope images of 60 min treated *S. cerevisiae* cultures with 16 μM BODIPY-HsAFP1. Propidium iodide (PI; 2 μg/mL) was used to identify membrane permeabilization. **(A)** Confocal images acquired with normal laser intensities (Alexa fluor 488-10% (argon laser)-HV573 and PI-1% (559 nm laser)-HV600). **(B)** Confocal images taken at high laser intensities [Alexa fluor 488-10% (argon laser)-HV700 and PI-1% (559 nm laser)-HV600], without impacting autofluorescence. Representative BODIPY-HsAFP1 accumulation spots at the cell surface are indicated with arrows. Bar: 5 μm.

To get more insight in BODIPY-HsAFP1’s localization, being at the cell surface and/or intracellularly, we performed additional confocal microscopy using an intermediate BODIPY-HsAFP1 dose (16 μM) and shorter treatment time (60 min). At normal laser intensities, only cells with intense green fluorescence signal were apparent (**Figure 7A**). These cells had permeabilized membranes and the BODIPY-HsAFP1 signal was intracellularly located. Additionally, at higher laser intensities, a subpopulation of PI- cells with BODIPY-HsAFP1 located at the cell surface, mainly at the buds and septa (as indicated by the arrows in **Figure 7B**) was apparent.

**FIGURE 8:**
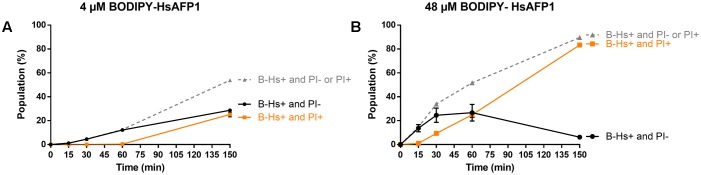
Kinetics of HsAFP1 internalization and membrane permeabilization in *S. cerevisiae* treated with **(A)** low (4 μM) or **(B)** high (48 μM) BODIPY-HsAFP1 (B-Hs) and 2 μg/mL propidium iodide (PI), determined via flow cytometry. The gray dashed line represents the percentage of B-Hs+ yeast cells in the population, relative to control (MQ water) treatment, which can be further divided in two subpopulations: yeast cells with (PI+; orange squares) and without (PI–; black circles) compromised membranes. Data are means ± SEM, for *n* = 3 experiments. To analyze significant differences in the size of the subpopulations between the time zero and other time points, one-way ANOVA followed by Dunnett multiple comparison was performed.

To quantify the different BODIPY-HsAFP1/PI populations over time in treated yeast cultures, we used flow cytometry (**Figure 8**). Treatment of yeast cultures with 48 μM BODIPY-HsAFP1 resulted in the appearance of a B-Hs+/PI- subpopulation after 15 min (14% of the treated culture), whereas a B-Hs+/PI+ subpopulation was apparent after 30 min (9% of the treated culture; *P* = 0.0007). Note that the B-Hs+ population might consist of cells that accumulate BODIPY-HsAFP1 on their surfaces and/or cells that accumulate BODIPY-HsAFP1 intracellularly. Treatment of yeast cultures with a low BODIPY-HsAFP1 concentration (4 μM) resulted in the appearance of a BODIPY-HsAFP1 (B-Hs)+/PI- subpopulation after 30 min (*P* = 0.0228), whereas a B-Hs+/PI+ subpopulation was apparent after 150 min (22% of the treated culture; *P* > 0.0001).

Together, the flow cytometry data showed that treatment of a yeast culture with BODIPY-HsAFP1 results first in accumulation of BODIPY-HsAFP1 at the cell surface and/or intracellularly (B-Hs+), after which membrane permeabilization occurs (PI+). Microscopic analysis indicated that BODIPY-HsAFP1 primarily associates with buds and septa, without affecting membrane integrity.

## Discussion

Similar as for some other plant defensins, we showed that HsAFP1 interacts with various lipids using lipid overlay assays (PIP Strips) (Sagaram et al., 2013; Baxter et al., 2015; Payne et al., 2016) as well as rELISA assays (Thevissen et al., 2004). Using PIP Strips, PtdIns(3,4,5)P_3_ was identified as the strongest interaction partner of HsAFP1 followed by PtdIns(3,4)P_2_. As PtdIns(3,4,5)P_3_ and PI(3,4)P_2_ are not present in yeast (Vanhaesebroeck et al., 2001), PA seems the main HsAFP1-interactor. These results were further confirmed in a more quantitative assay (rELISA setup), also indicating PA as the strongest of the potential interaction partners of HsAFP1 tested. As both assays can give an indication of lipid specificity but lack biological environment, we additionally tested HsAFP1-PA interactions in a membrane context. We showed that HsAFP1 specifically interacted with vesicles containing PA. However, as these interactions could only be observed when using low salt concentrations, this interaction is presumably mediated via electrostatic binding. Moreover, as the area under the melting curve did not change by HsAFP1 addition, this interaction seems superficial. In addition, using rELISA assays, we demonstrated that HsAFP1 reversibly binds to PA, which is consistent with our previous findings on HsAFP1-membrane target interactions (Thevissen et al., 1997). We further identified the region in HsAFP1 responsible for PA binding via rELISA assays in which competition between HsAFP1 and HsAFP1-derived peptide fragments for PA binding was assessed. We found that the C-terminal domain of HsAFP1 (HsLin06) can prevent binding of HsAFP1 to PA, indicating that this domain in HsAFP1 mediates PA binding. Moreover, the arginine (R) at position 52 in HsAFP1 seems essential for PA binding, while the histidine (H) at position 32 in HsAFP1 seems favorable, but not essential for PA binding. Both amino acids are located in close proximity of each other on the HsAFP1 structure (green cluster in **Figure 3D**). The other positively charged amino acids in the C-terminal part of HsAFP1 (H40, lysine (K) 47 and K51), which seem not important for PA binding, are differently located (red cluster in **Figure 3D**). This is in line with reports of other proteins that bind to PA, such as mTOR, in which multiple amino acids contribute to PA docking, with an arginine being necessary for this interaction (Veverka et al., 2008). Hitherto, three other plant defensins that interact with PA have been identified, namely NsD7 (Kvansakul et al., 2016), MtDef4 (Sagaram et al., 2013) and NaD2 (Payne et al., 2016). However, neither primary sequence homology nor structural alignments were found between the PA-interacting RGFRRR region (located in the γ-core) in MtDef4 (Sagaram et al., 2013), which is also present in NaD2 (Payne et al., 2016), and the corresponding region in HsAFP1 or NsD7 (**Figure 2**). A common feature of the PA binding region of HsAFP1, NsD7, MtDef4 and NaD2 is the presence of positively charged amino acids. This is in line with previous reports on the amino acid sequences of various PA effectors that indicated that there is no defined PA binding sequence, and only the presence of both positively charged amino acids and hydrophobic amino acids can enable PA binding in lipid environments (Stace and Ktistakis, 2006; Shin and Loewen, 2011).

Since the phosphomonoester (phosphate) head group of PA is negatively charged and therefore mainly responsible for electrostatic interactions with basic amino acid residues of PA effector proteins, we examined HsAFP1 interaction with methyl-PA, which bears a methyl-group substitution on the phosphate moiety (Young et al., 2010), making the latter less negatively charged and less available for binding. As expected, this resulted in a reduction of HsAFP1 binding by more than 75%. Moreover, besides the interaction of HsAFP1 with the phosphate group of PA, we demonstrated that free phosphates in the buffer can compete with PA for HsAFP1 binding, pointing to phosphate-HsAFP1 interactions.

Besides PA, HsAFP1 also bound to various other phospholipids, as was previously shown for MsDef1, MtDef4, NaD1 and NaD2 (Sagaram et al., 2013; Baxter et al., 2015; Payne et al., 2016). Moreover, we demonstrated for the HsAFP1[H32A][R52A] double mutant that H32 and R52 are important for PtdInsP binding as well. This is not surprising as different PA effectors have also been shown to bind to other lipids, such as PtdInsP’s (Testerink and Munnik, 2011). In case of HsAFP1, it seems that the phosphate group(s) present in PA and all PtdInsP’s, enable(s) interactions with HsAFP1, since lipids containing at least one phosphate group were identified as HsAFP1 interaction partners in the PIP Strip overlay assays. Why HsAFP1 binds preferentially with PA in the rELISA assays has to be elucidated further. As shown for the PA-interacting plant defensin NsD7 (Kvansakul et al., 2016), it might be that HsAFP1’s PA-binding site contains a small pockets size that can only accommodate a single phosphate group, which makes it selective for PA. Consistent with our data, it has been reported that NaD1 interacts with phosphate groups of PtdIns(4,5)P_2_. This leads to oligomerization of NaD1:PtdIns(4,5)P_2_ complexes (Poon et al., 2014), which was recently found not essential for the antifungal activity of NaD1 (Bleackley et al., 2016).

Most importantly, using the HsAFP1[H32A][R52A] double mutant, we showed that phospholipid (PA and PtdInsP’s) interacting capacity of HsAFP1 is important for its antifungal activity. Based on our microscopic and flow cytometry analyses, we propose a 3-step killing process of the plant defensin HsAFP1 in *S. cerevisiae*. Firstly, HsAFP1 accumulates in low amounts at the yeast cell surface and this accumulation is most pronounced at the buds and septa. It has been shown that PtdIns(4,5)P_2_ is enriched in septa, as this phospholipid is the main regulator of cytokinesis (Brill et al., 2011). Next, HsAFP1 is internalized in yeast cells, immediately followed by membrane permeabilization as only a very small subpopulation (<1%) of HsAFP1-treated yeast cells exist that show internalization of HsAFP1 with intact membranes. After permeabilization, additional BODIPY-HsAFP1 might diffuse further into the cells, resulting in cells with a very intense green fluorescence signal, as was apparent in microscopy performed at high BODIPY-HsAFP1 doses after 150 min of incubation. We previously demonstrated that HsAFP1 treatment of a yeast culture results in a subpopulation of apoptotic cells (i.e., dead cells that are PI-) (Aerts et al., 2011). In the current study, on average 10% of the HsAFP1-treated cultures was found dead, as determined via plating assays (data not shown), while being PI- (based on the corresponding flow cytometry analysis of the cultures). Whether these apoptotic cells internalized HsAFP1 is not clear. Hence, future work in our laboratory will be directed to the use of a systems biology approach to investigate the kinetics of BODIPY-HsAFP1 internalization and cell death, thereby also focusing on yeast apoptosis, using single cell digital microfluidics (DMF).

Similar to HsAFP1, peptide accumulation on the cell surface prior to membrane permeabilization and cell entry has been demonstrated for the plant defensin NaD1 (Hayes et al., 2013). As HsAFP1 internalization is probably not resulting from the HsAFP1-induced membrane permeabilization in *S. cerevisiae*, peptide translocation across cell membranes independent of membrane permeabilization should be part of HsAFP1 killing mechanism. Pore-independent peptide translocation has been reported for some antimicrobial peptides (AMPs) and is thought to be mediated by either endocytic or physical (spontaneous protein-membrane interaction) processes. Henriques and colleagues suggested that depending on the circumstances (such as peptide concentration and temperature), AMPs can be internalized via both routes (Henriques et al., 2006). We found that HsAFP1’s internalization is at least partially mediated by endocytosis as it was significantly reduced in a yeast mutant that is affected in endocytosis (*Δend3*) (Raths et al., 1993) (data not shown).

## Conclusion

We showed that HsAFP1 can bind to various lipids which contain at least one phosphate group, with PA being the lipid binding partner resulting in the most pronounced HsAFP1 binding. We identified two basic amino acids in HsAFP1, the histidine at position 32 and arginine at position 52, as well as the phosphate group in PA as important features enabling this interaction. Hence, we can conclude that the RGFRRR region, present in the PA-interacting plant defensins MtDef4 and NaD2 and previously defined as the region important for PA binding and cell entry, is not the only domain enabling PA binding of plant defensins, as this region is not present in HsAFP1. Moreover, we showed that HsAFP1 is accumulating at the yeast cell surface, primarily in buds and septa, which results in membrane permeabilization, potentially upon internalization of the peptide.

## Author Contributions

Experiments were designed by TC, KV, BK, AT, JD, BC, and KT; KT coordinated the study. Lipid overlay assay were performed by TC and CS; rELISA assays and flow cytometry experiments were performed by TC; HsLin peptides were synthesized by JD, vesicle assays were performed by TC, MR, GB, and CB; confocal microscopy was done by TC, LD, SK, and PVD. Statistical analysis were performed by TC and SV. The drawing in **Figure 3D** was made by TC. The manuscript was written by TC and KT; and revised by KV, CS, SV, MR, GB, CB, BK, AT, JD, LD, SK, PVD, DS, JL, and BC. All authors have read and approved the final manuscript.

## Conflict of Interest Statement

The authors declare that the research was conducted in the absence of any commercial or financial relationships that could be construed as a potential conflict of interest. The reviewer LN and handling Editor declared their shared affiliation.
