# Supplementary material for: The Antifungal Plant Defensin HsAFP1 Is a Phosphatidic Acid-Interacting Peptide Inducing Membrane Permeabilization
Source: Front Microbiol. 2017 Nov 21;8:2295. doi: 10.3389/fmicb.2017.02295 (PMC5702387; doi:10.3389/fmicb.2017.02295)
Supplement: Supplementary file 1 [file Data_Sheet_1.doc]

***Supplementary Material***

**The antifungal plant defensin HsAFP1 is a phosphatidic acid-interacting peptide inducing membrane permeabilization**

**Tanne L. Cools, Kim Vriens, Caroline Struyfs, Sara Verbandt, Marcelo H. S. Ramada, Guilherme D. Brand, Carlos Bloch Jr., Barbara Koch, Ana Traven, Jan W. Drijfhout, Liesbeth Demuyser, Soňa Kucharíková, Patrick Van Dijck, Dragana Spasic, Jeroen Lammertyn, Bruno P. A. Cammue^*^ and Karin Thevissen**

*** Correspondence: Bruno P. A. Cammue:** [bruno.cammue@kuleuven.be](mailto:bruno.cammue@kuleuven.be)

**Supplementary Figure S1: Reversible HsAFP1 – phosphatidic acid (PA) binding, determined via sequential rELISA assays.** Competition of 12.5 µM HsAFP1 with an excess (4x) of HsLin06 for PA binding, with HsAFP1 administered 30 min prior to HsLin06. Data are means ± SEM, for n ≥ 3 experiments. Data are expressed relative to the HsAFP1-PA binding (black bar). Significant differences between HsAFP1 and HsAFP1 + 4x HsLin06 interactions were determined via an unpaired student t-tests with Welch’s correction, with ** representing P < 0.01.


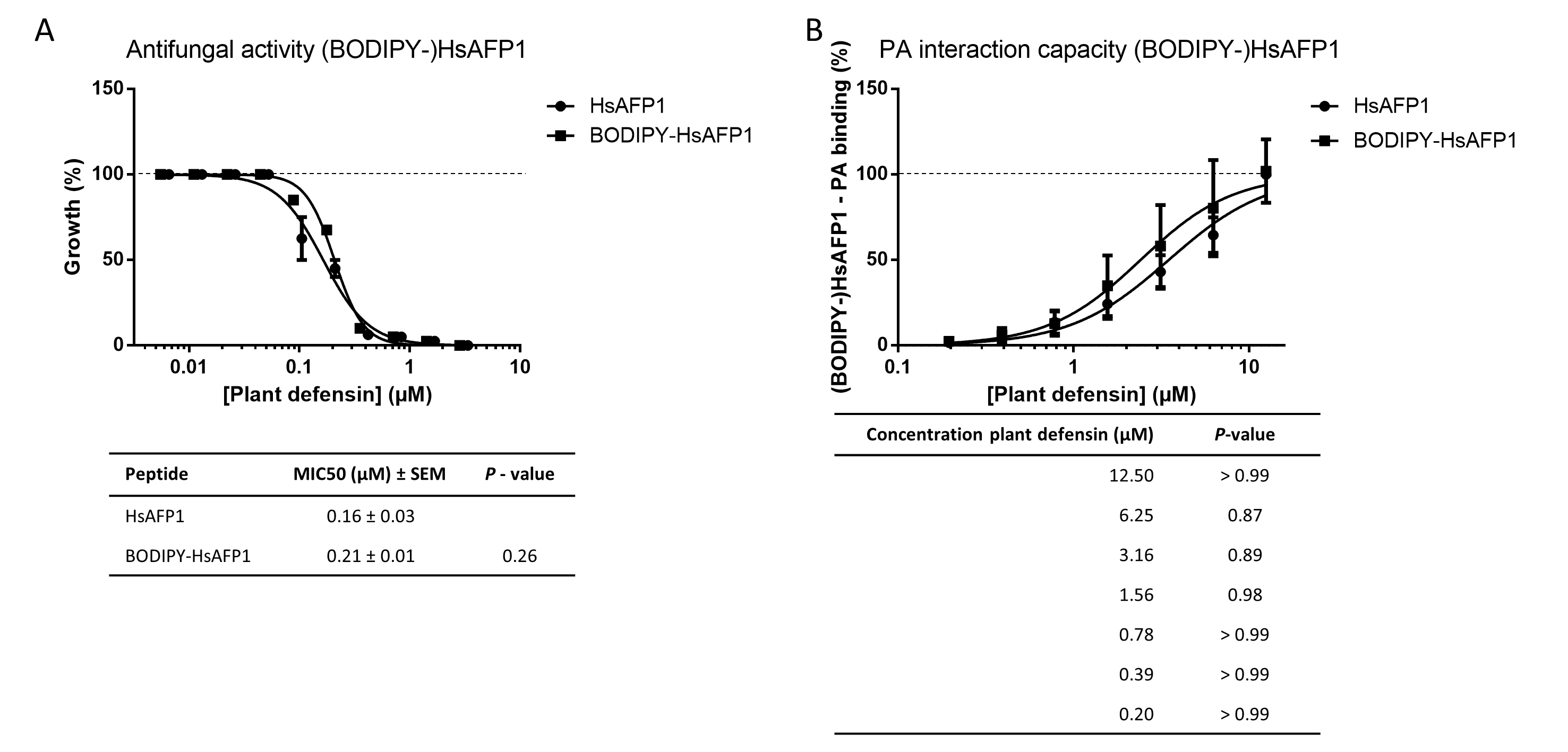


**Supplementary Figure S2: BODIPY labelling of the plant defensin HsAFP1 did not affect antifungal activity nor PA interaction capacity.** Data were means ± SEM, for n = 2 experiments. (**A**) Dose-response curves of the antifungal activity of (BODIPY-)HsAFP1 on Fusarium culmorum. MIC50, Minimum Inhibitory Concentration required for 50% growth inhibition compared to control (MQ water) treatment (dotted line). An unpaired student t-test was performed to analyse significant differences in MIC50 values between HsAFP1 and BODIPY-HsAFP1, with P < 0.05 is significant. (**B**) Dose-response curves of (BODIPY-)HsAFP1 binding on PA, with all data expressed relative to the condition with the highest HsAFP1 binding (dotted line). For each plant defensin concentration significant differences were determined via two-way ANOVA followed by Sidak multiple comparison, with P < 0.05 is significant.


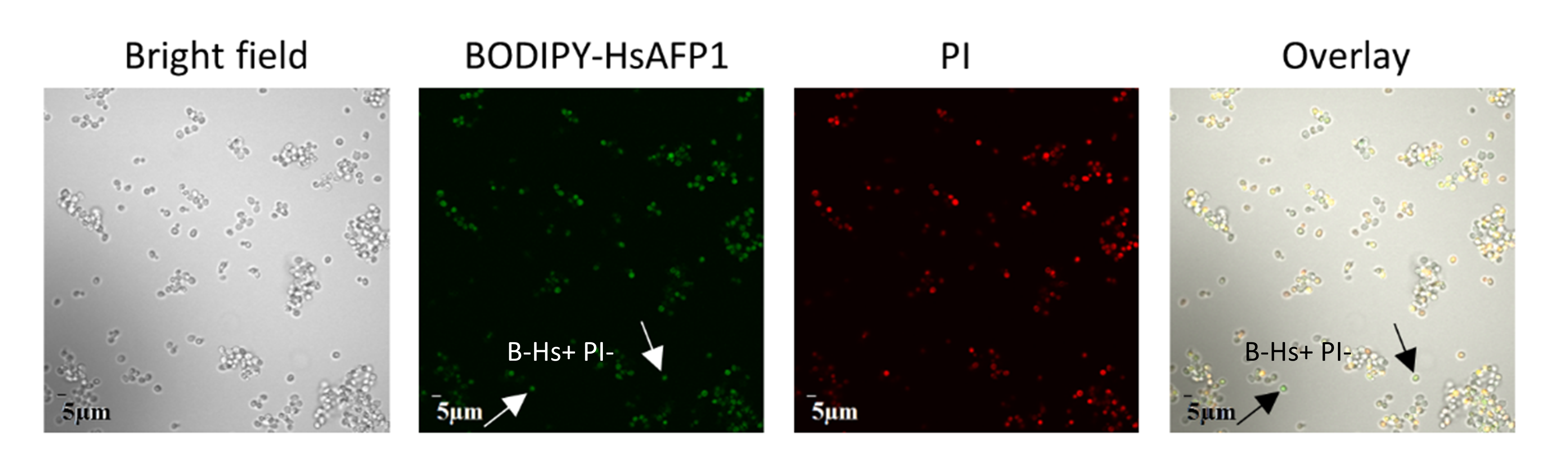


**Supplementary Figure S3: BODIPY-HsAFP1 (B-Hs) internalization in *S. cerevisiae* cells with intact membranes.** Confocal microscope images of 150 min treated *S. cerevisiae* cultures with 48 µM BODIPY-HsAFP1 and propidium iodide (PI; 2 µg/mL) to identify membrane permeabilization. Representative highly intense coloured B-Hs cells with intact membranes (B-Hs+/PI-) are indicated with arrows.
